# Supplementary material for: Convergent use of phosphatidic acid for hepatitis C virus and SARS-CoV-2 replication organelle formation
Source: Nat Commun. 2021 Dec 14;12:7276. doi: 10.1038/s41467-021-27511-1 (PMC8671429; doi:10.1038/s41467-021-27511-1)
Supplement: Supplementary file 1 — Supplementary Information [file 41467_2021_27511_MOESM1_ESM.pdf]

Supplementary Information

Convergent use of phosphatidic acid for Hepatitis C virus and SARS-CoV-2 replication organelle formation

Keisuke Tabata*, Vibhu Prasad*, David Paul, Ji-Young Lee, Minh-Tu Pham, Woan-Ing Twu, Christopher J. Neufeldt, Mirko Cortese, Berati Cerikan, Yannick Stahl, Sebastian Joecks, Cong Si Tran, Christian Lüchtenborg, Philip V'kovski, Katrin Hörmann, André C. Müller, Carolin Zitzmann, Uta Haselmann, Jürgen Beneke, Lars Kaderali, Holger Erfle, Volker Thiel, Volker Lohmann, Giulio Superti-Furga, Britta Brügger, and Ralf Bartenschlager [&]

* These authors contributed equally to this work
[&] Correspondence to: ralf.bartenschlager@med.uni-heidelberg.de

This PDF file includes:

Supplementary Figs. 1 to 10
Supplementary Tables 1 to 5
References
Source Blots

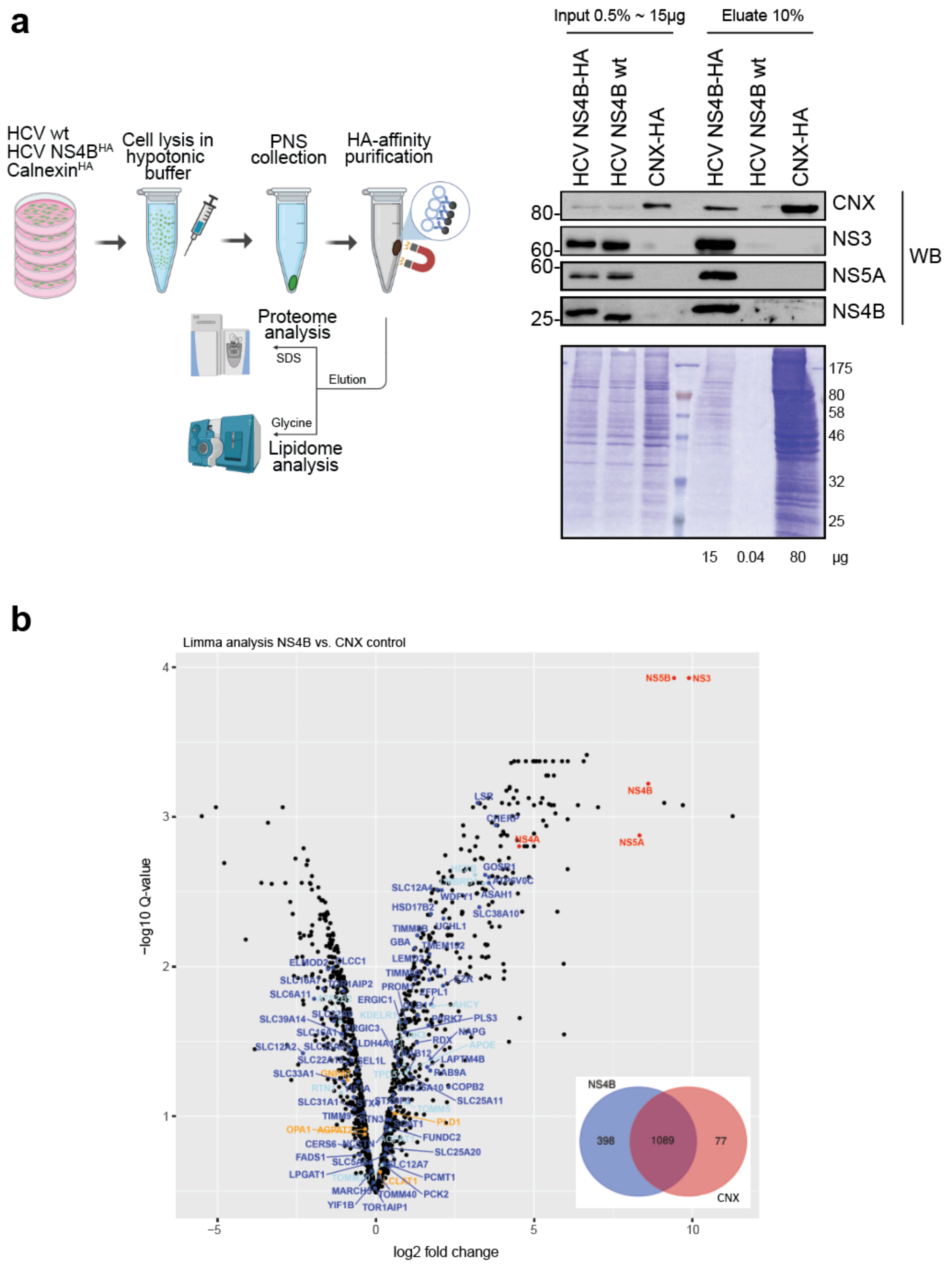

Supplementary Figure 1. Purification and proteome of NS4B-associated membranes. a NS4B-associated DMV fractions were isolated from Huh7-Lunet cells containing a stable subgenomic HCV replicon of the isolate JFH1. This replicon encoded either wildtype or an HA-tagged NS4B (NS4B-wt and NS4B-HA, respectively). Huh7-Lunet cells stably expressing HA-tagged calnexin (CNX-HA) were used as biological reference. Cell lysates generated by hypotonic lysis were centrifuged and post-

nuclear supernatants (PNS) were applied to affinity purification using magnetic beads coated with an HA-specific antibody. After extensive washing of lysate-loaded beads, captured complexes were eluted with SDS (for proteome analysis) or glycine [pH 2.5] (for lipidome analysis). Given amounts of cell lysate (input) and eluate were analyzed by western blotting (top panel) and Coomassie blue staining (bottom panel). Numbers on the bottom right refer to total protein yield in the given fractions (in µg). Molecular weight markers (in kDa) are indicated on the left of the Western blot and on the right of the Coomassie blue stained gel. Samples of all three conditions were prepared in duplicates and analyzed by LC-MS/MS based proteomics. **b** Volcano plot of differentially enriched interactors of NS4B and CNX. The q-value was calculated using the limma R software package[1] and corrected for multiple hypothesis testing. Proteins selected for validation are highlighted by dark blue, proteins confirmed by siRNA screen in orange and virus proteins in red color.

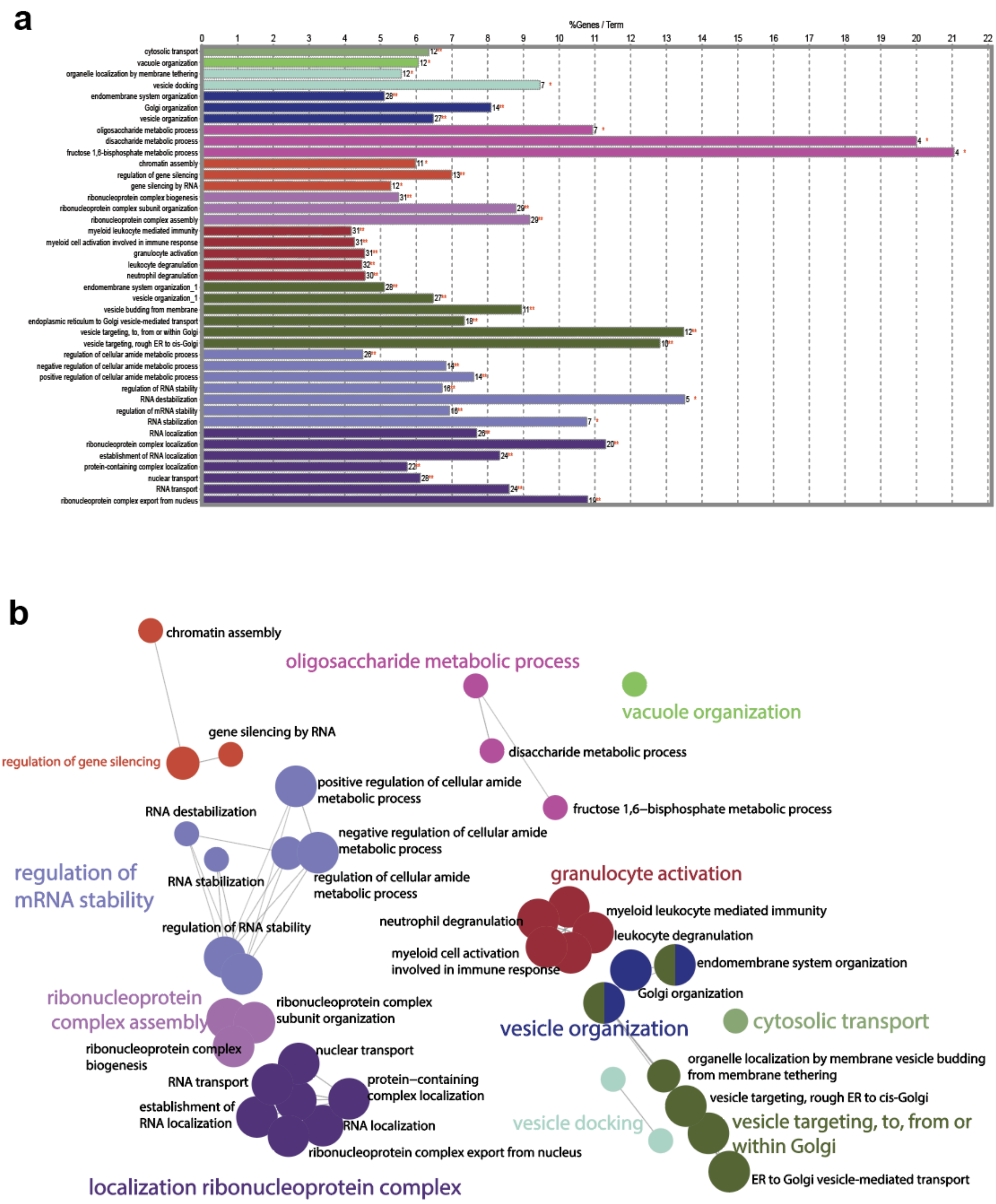

Supplementary Figure 2. Gene ontology analysis of proteome hits identified in NS4B-enriched membrane fractions. List of 309 proteins showing a ≥2-fold enrichment in NS4B-associated DMV fraction versus HA-tagged calnexin (CNX-HA) and having limma statistical q-values higher than 0.05 were used for gene ontology (GO) analysis. Cytoscape 3.7.2[2] and ClueGO[3] app were used to generate and visualize non-redundant biological terms for large clusters of protein accessions (human uniprot accession IDs) in a functionally grouped network. ClueGO results are illustrated as a functionally grouped network of terms. **a** Bar chart of ClueGO significant biological functional terms organized in multiple term occurrences. Numbers represent protein accessions per term. **b** ClueGO results represented as functionally grouped network of terms with node size showing significance of cluster.

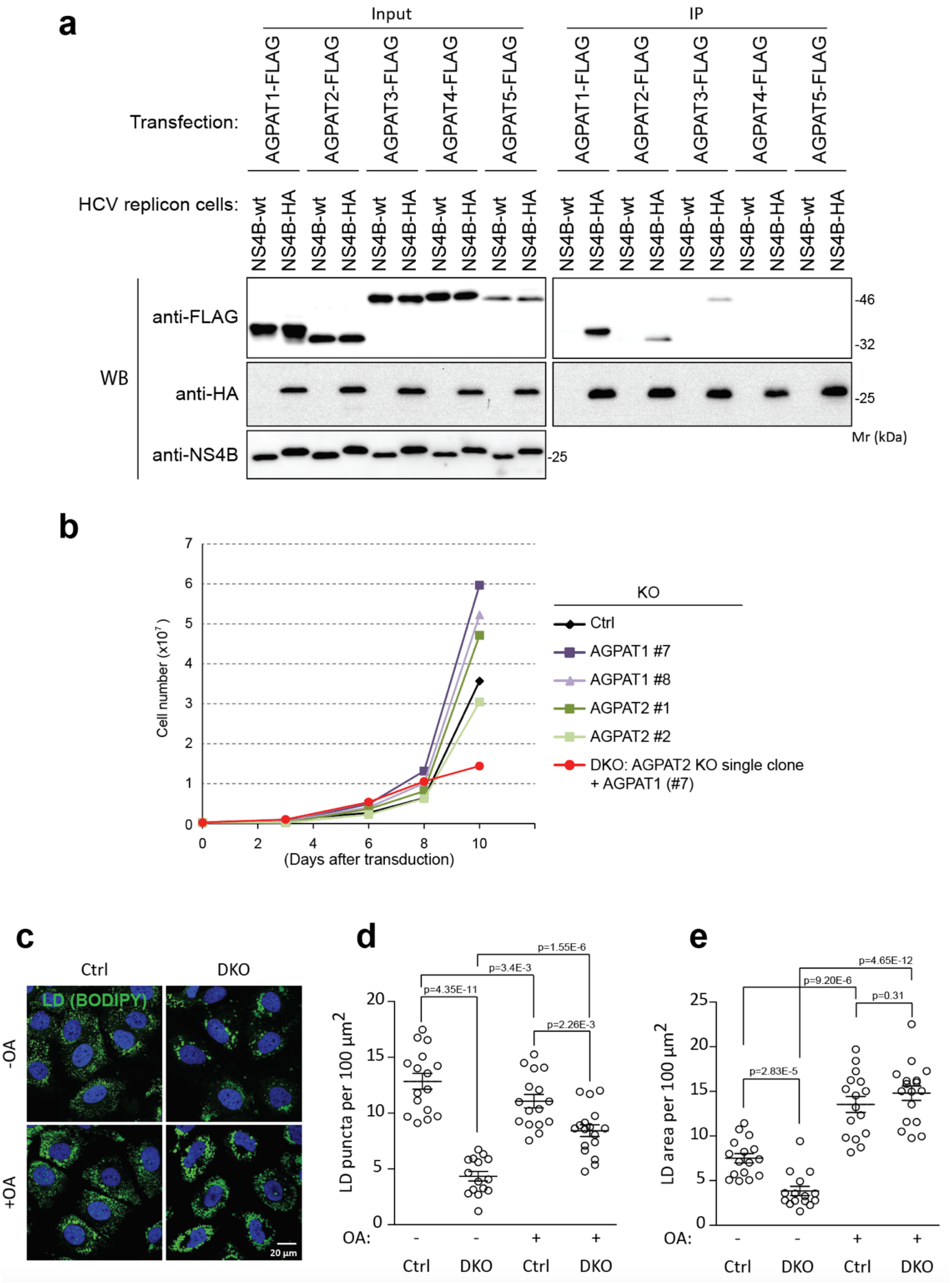

Supplementary Figure 3. Interaction of NS4B with AGPAT isoforms and impact of single and double AGPAT1/2 knock-out on cell viability as well as lipid droplet formation. a Huh7-Lunet cells containing a stably replicating HCV replicon with wildtype or HA-tagged NS4B were transfected to express FLAG-tagged AGPATs. Two days after transfection NS4B-HA enriched membrane fractions were prepared under native conditions by HA-specific immunoprecipitation (IP). Proteins in

lysates (1% of input) and captured protein complexes (20% of eluate) were analyzed by western blotting (WB). Three biologically independent experiments showed similar results. **b** Effect of AGPAT1/2 single and double knock-out on cell growth. Cells were infected with sgRNA encoding lentivirus on day-0 and expanded until day-10 using increasingly bigger culture dishes according to cell growth. Cell numbers were determined by using an automated cell counter. The experiment was repeated twice. **c-e** Control KO and AGPAT1/2 DKO cells were incubated without or with 50 µM oleic acid-BSA (-OA and +OA, respectively) for 16 h. **c** Representative images. Lipid droplets (LD) and nuclear DNA were stained with BODIPY 493/503 (green) or DAPI (blue), respectively. **d, e** The number of LD puncta per 100 μm^2 cell surface area (d) or total LD area per 100 μm^2 (e) were determined and are given as average and SEM. n= more than 15 cells examined over two independent experiments. Significance was calculated by two-tailed paired t-test and p-values are indicated in the figure. Source data for panels a, b, d, e are provided as Source Data file.

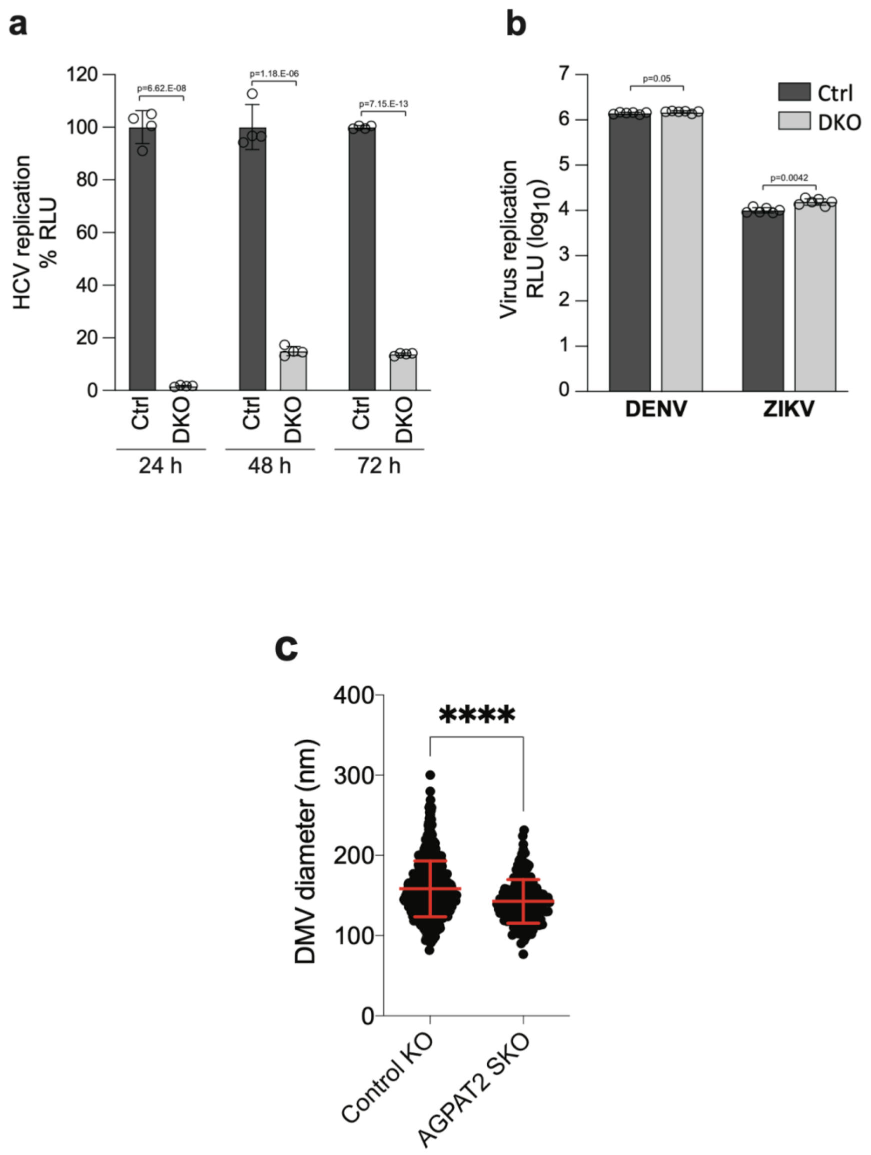

Supplementary Figure 4. Effect of AGPAT1/2 DKO on replication and DMV formation of HCV and on replication of other flaviviruses. a Huh7-Lunet/T7 cells were electroporated with in vitro transcripts of a subgenomic HCV reporter replicon encoding the firefly luciferase. Luciferase activities were analyzed at indicated time points after electroporation. Graph shows average and SD from 4 independent experiments. Significance was calculated by one-tailed paired t-test. p values are shown in the graph. **b** AGPAT1/2 DKO does not impair DENV and ZIKV replication. Cells were infected with DENV or ZIKV *renilla* luciferase reporter viruses and 48 h later, RNA replication was determined by luciferase assay. Values are expressed as average of RLU (log_{10}) and SD from 6 independent experiments. Significance was calculated by one-tailed paired t-test. p values are shown in the graph. **c** Reduced HCV DMV diameter in AGPAT2 single (S)KO cells. Huh7-derived cells stably expressing the T7 RNA polymerase and without or with SKO of AGPAT 2 were transfected with the HCV replicase-encoding plasmid containing a GFP insertion in NS5A (construct pTM NS3-5B/5A-GFP)[4]. After 24 h, cells were fixed and subjected to CLEM. DMV diameters within whole cell sections were counted and plotted. Data are represented as mean ± SD. n= more than 174 DMVs from 5 cell sections. Significance was calculated by two-tailed paired t-test. ****, p=6.72597E-8. Source data for panels a, b, c are provided as Source Data file.

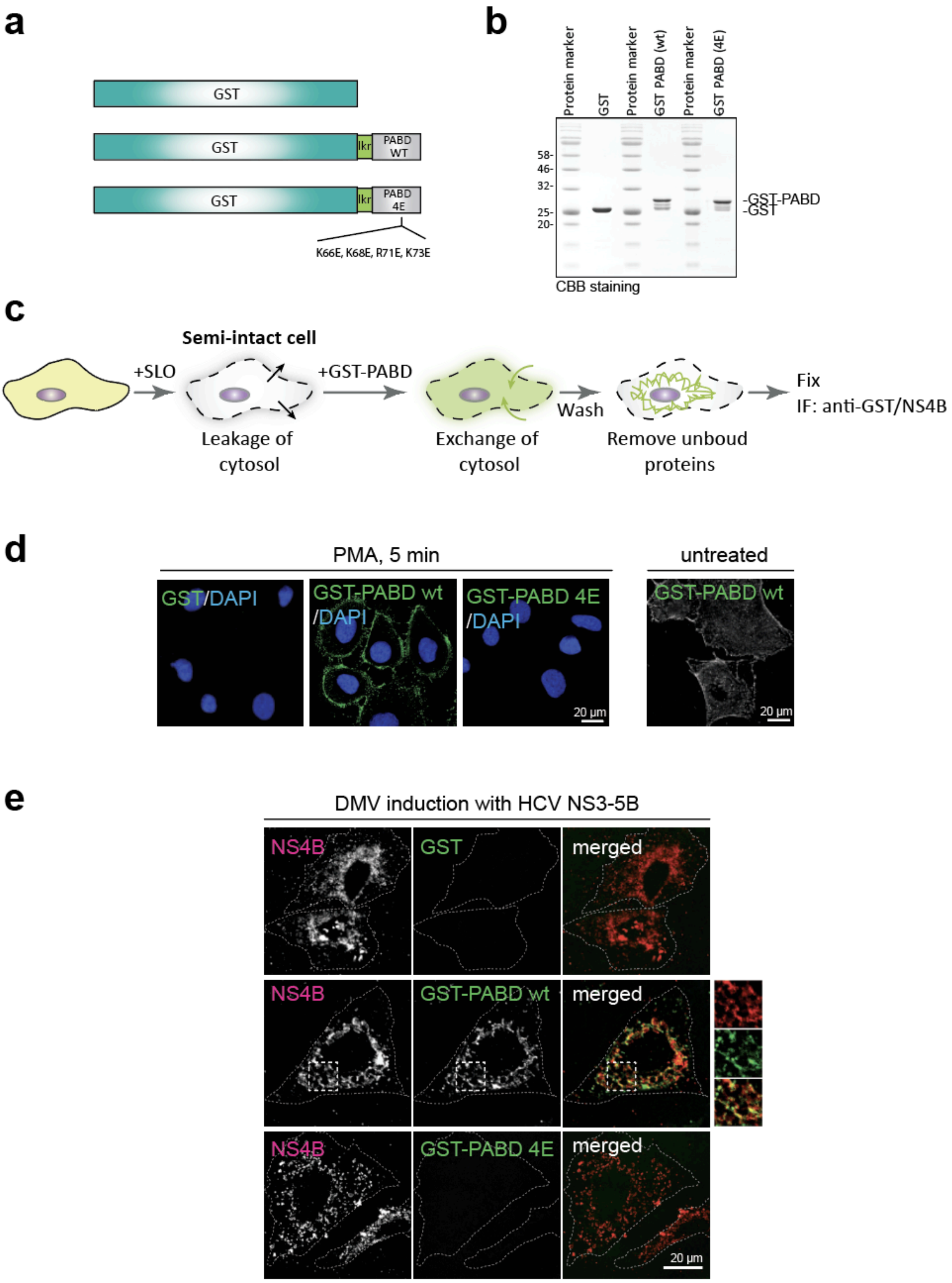

Supplementary Figure 5. Subcellular PA distribution in HCV replicase-expressing cells as determined with a recombinant PA sensor. a Schematic of the recombinant PA-binding protein that is composed of the glutathione S-transferase (GST) fused to the PA binding domain (PABD) of the Spo20 protein via a short linker (lkr). The 4E mutant contains 4 amino acid substitutions specified on the bottom. **b** Fusion proteins were expressed in E. coli and pre-purified cell lysates were subjected to GST-specific affinity chromatography. Five microgram recombinant protein were loaded onto a SDS-polyacrylamide gel that was stained with coomassie brilliant blue (CBB) after electrophoresis. Two

biologically independent experiments showed similar results. **c** Experimental approach to visualize PA with the exogenously added recombinant biosensor. Cells were transiently permeabilized by treatment with streptolysin O (SLO) that forms pores in the plasma membrane and allows the partial exchange of the cytosol against a physiological solution containing the purified recombinant PA biosensor. Bound GST-PABD and NS4B were visualized by immunofluorescence (IF) microscopy using GST- and NS4B-specific antibodies. **d** Functionality of the recombinant PA sensor. Huh7-Lunet/T7 cells were treated with 100 nM PMA for 5 min or left untreated and PA was visualized by IF using recombinant proteins specified on the top of each panel. Nuclear DNA was stained with DAPI to visualize all cells on the coverslip. Three biologically independent experiments showed similar results. **e** Huh7-Lunet/T7 cells were transfected with the HCV NS3-5B encoding plasmid, followed by permeabilization with SLO and addition of purified GST-PABD wt or 4E proteins. Bound proteins were detected by GST-specific immunofluorescence microscopy. Three biologically independent experiments showed similar results.

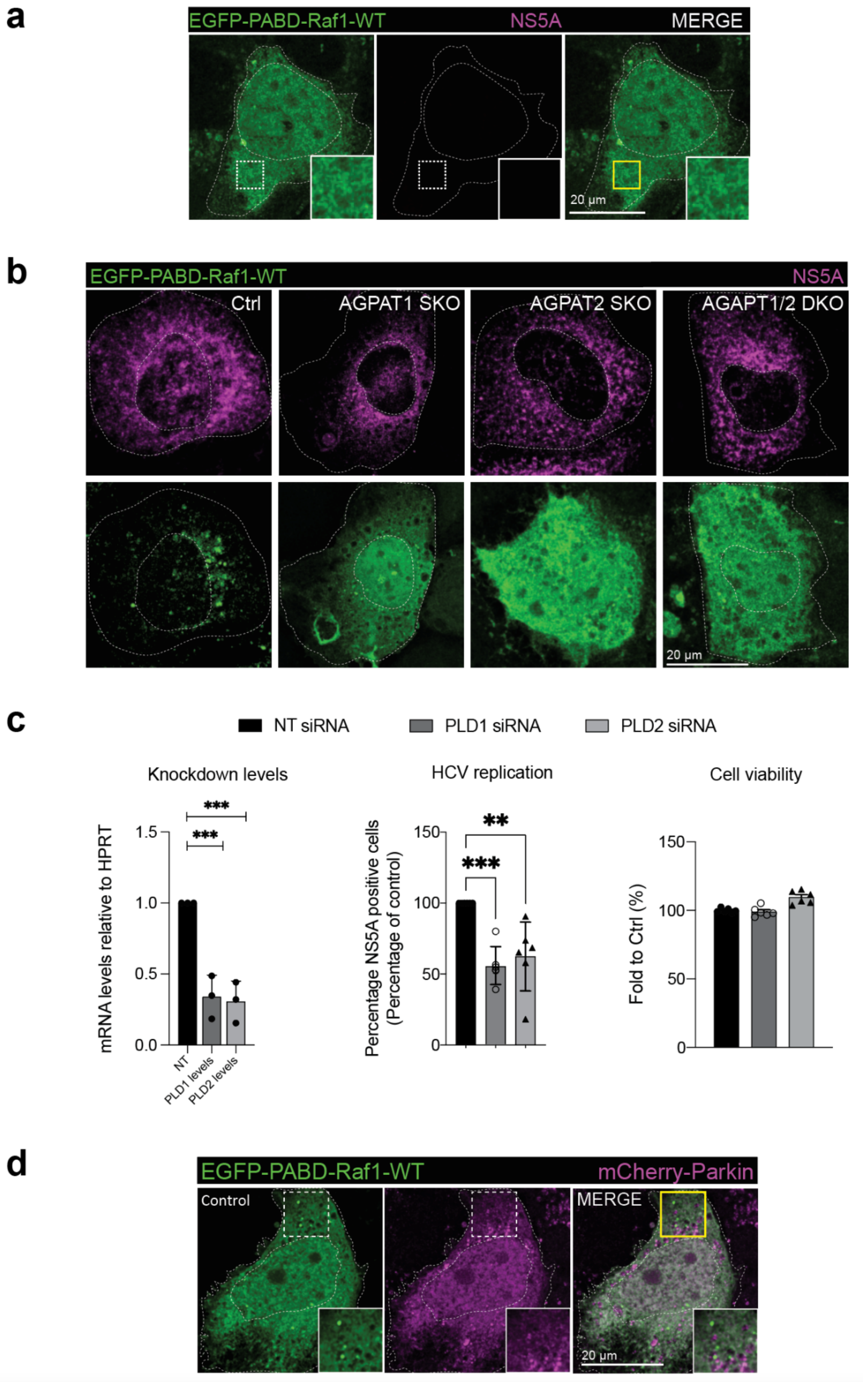

Supplementary Figure 6. PA distribution in naïve Huh7-derived cells. a Huh7-Lunet/T7 cells were transfected with a construct encoding EGFP-tagged wildtype (WT) PA sensor (construct pTM-EGFP-PABD-Raf1-WT). Twenty-four hours later, cells were fixed and GFP-PABD was visualized by

fluorescence microscopy. White boxes indicate regions magnified in the lower right of each panel. Data shown here belong to the results shown in Fig. 4a and they reveal a diffuse PA-sensor distribution in the absence of HCV NS3-5B expression. Two biologically independent experiments showed similar results. **b** Huh7-Lunet control, AGPAT2 single (SKO) and AGPAT1/2 double (D)KO cells were cotransfected with constructs encoding an EGFP-tagged wildtype (WT) PA sensor (construct pTM-EGFP-PABD-Raf1-WT) and HCV NS3-5B with NS5A having an in-frame insertion of mCherry (construct pTM NS3-3'/5A-mCherry). Twenty-four hours later, cells were fixed and EGFP-PABD and NS5A-mCherry were visualized by fluorescence microscopy. A representative result of three biologically independent experiments is shown. **c** PLD1 and PLD2 knock-down reduces HCV replication. Huh7.5 cells were transfected with siRNAs targeting PLD1 and PLD2 and 48 h later infected with HCV (strain Jc1; MOI=1). Twenty-four hours later, cells were fixed, immunostained for HCV NS5A and percentage of positive cells was quantified using CellProfiler. Normalized data from three biologically independent experiments are plotted. Data are represented as mean ± SD. **d** Subcellular distribution of the PA-sensor and mCherry-Parkin in the absence of mitophagy. Huh7-derived cells were co-transfected with EGFP-PABD-Raf-1 and mCherry-tagged Parkin and 24 h later cells were fixed and analyzed by fluorescence microscopy. Two biologically independent experiments showed similar results. Data shown here belong to the results shown in Fig. 4c.

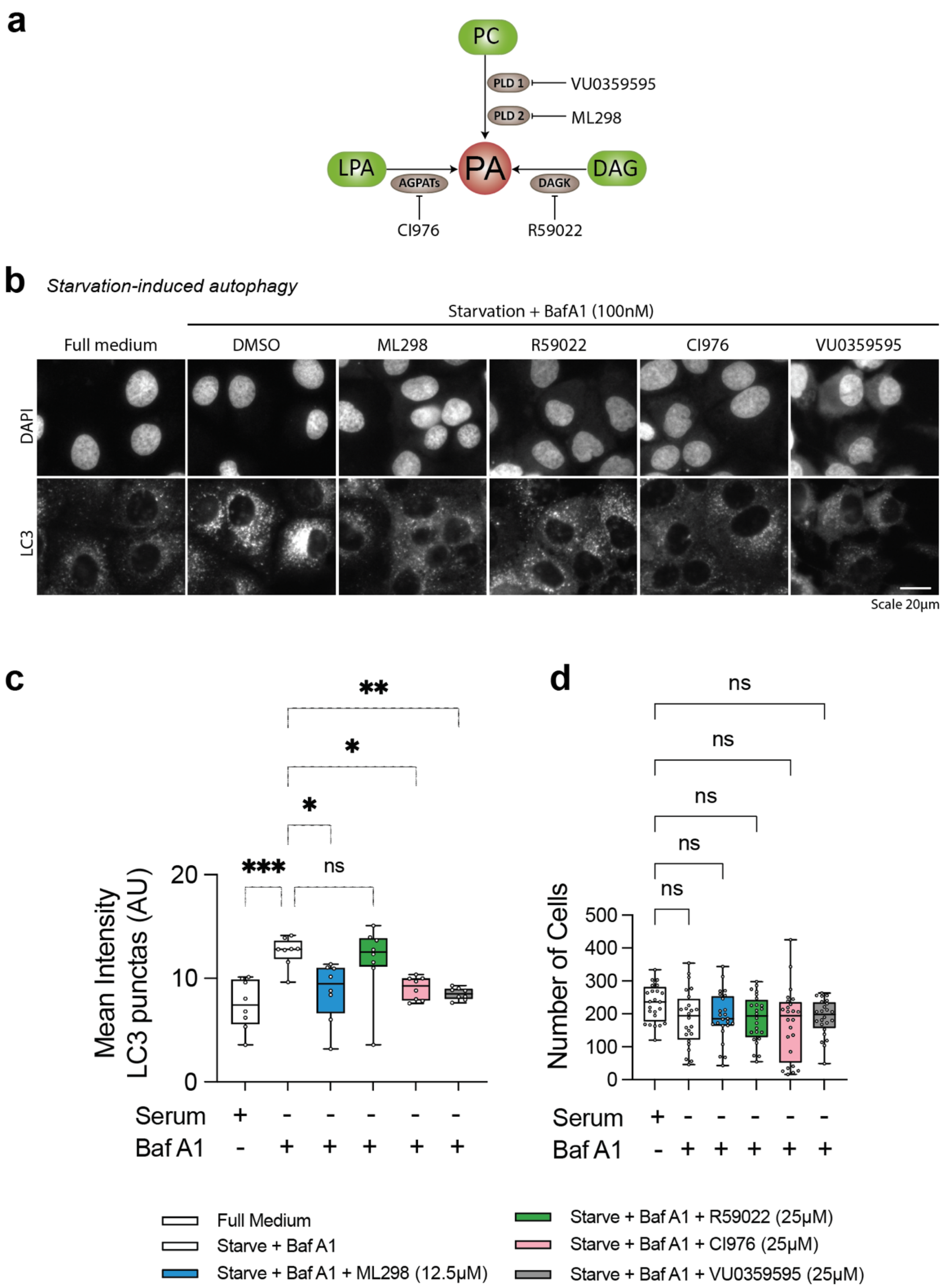

Supplementary Figure 7. Inhibition of PA production through PLD1, PLD2 and AGPATs decreases LC3 accumulation during nonselective autophagy. a PA biosynthesis pathways via lysophosphatidic acid (LPA), phosphatidylcholine (PC) and diacylglycerol (DAG), metabolized by AGPATs, PLDs and DAGK, respectively. **b** Effect of PA inhibitors on LC3 puncta. Huh7-Lunet/T7 cells were incubated in full medium or starvation medium containing BafA1, in the presence of PA inhibitors specified above the panels. After 3 h incubation, cells were fixed and stained with an LC3-

specific antibody followed by immunofluorescence microscopy. Nuclear DNA was stained with DAPI. Solvent (DMSO) treated cells served as control. **c** Quantification of mean LC3 puncta intensity per cell, and **d** total cells analyzed per condition, as determined by using a custom-made CellProfiler script that segments the LC3 puncta, nuclei and cell boundary and measures the intensities of LC3 puncta in the cytoplasmic area of single cells, indicated as a scatter plot with N=4000 cells per condition. The central line of the plot indicates median with first and third quartiles are shown as boxes, and whiskers as lines. Two biologically independent experiments gave similar results. Significance was calculated using ordinary one-way ANOVA. *, p=0.0243 (ML298) and 0.0480 (CI976); **, p=0.0078; ***, p=0.0009; ns, p=0.99.

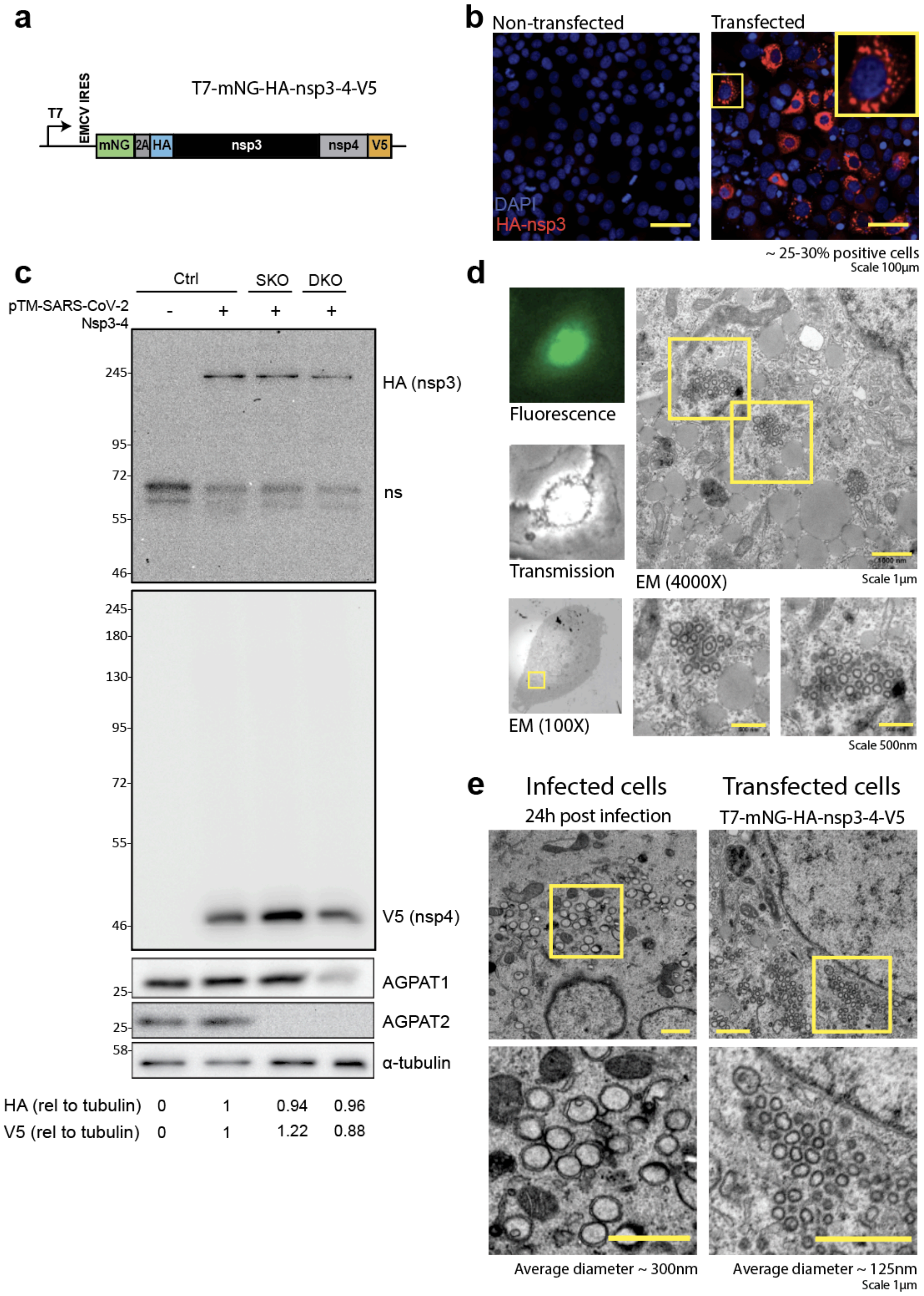

Supplementary Figure 8. SARS-CoV-2 nsp3-4 induced DMVs are morphologically similar to infection induced DMVs, and effect of AGPAT KO on viral protein expression. **a** Schematics of the T7-based expression construct encoding SARS-CoV-2 HA-nsp3-4-V5. mNG, NeonGreen. **b** Huh7-

derived cells were transfected with the SARS-CoV-2 HA-nsp3-4-V5 encoding plasmid and 24 h later cells were stained with an HA-specific antibody and analyzed by confocal microscopy to visualize HA-nsp3. Transfection efficiency given on the lower right was determined by cell counting. A magnification of the yellow boxed area is shown on the top right. Two biologically independent experiments showed similar results. **c** Abundance of SARS-CoV-2 proteins in transfected cells was quantified by western blotting using primary antibodies specified on the right of each panel. α-tubulin served as loading control. Data in **c** belong to the results shown in Fig. 6c and d. ns, non-specific band. Two biologically independent experiments showed similar results. **d** CLEM for quantification of nsp3-4 induced DMVs. Huh-derived cells stably expressing the T7 RNA polymerase were transfected with the HA-nsp3-4-V5 encoding plasmid, mNeonGreen positive cells were identified by fluorescence microscopy and selected for transmission electron microscopy. Yellow boxed regions are highlighted on the right panel and the two panels below, respectively. Two biologically independent experiments showed similar results. **e** Comparison of HA-nsp3-4-V5 induced DMVs (as in d) with DMVs induced in SARS-CoV-2 infected Calu-3 cells as determined by transmission electron microscopy. Average diameters of DMVs are given on the bottom. Diameter calculations are based on the analysis of at least 10 infected cells (as reported earlier[5]) or ~1650 DMVs detected in transfected cells. Source data for panel c are provided as Source Data file.

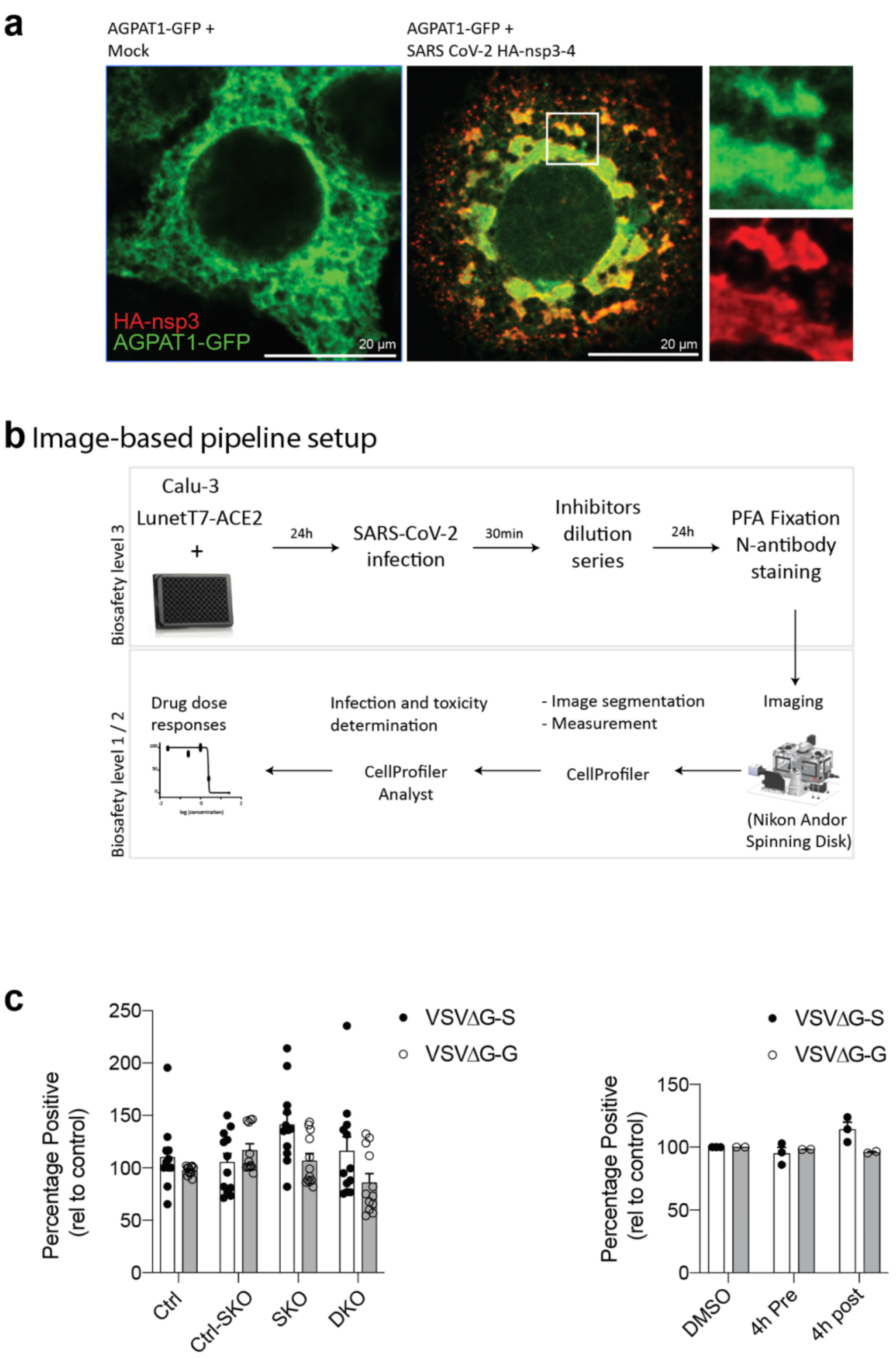

Supplementary Figure 9. AGPATs are re-localised to SARS-CoV-2 nsp3 containing structures, imaging based pipeline to quantify viral replication and role of AGPAT in SARS-CoV-2 entry. a Huh7-derived cells were co-transfected with AGPAT1-EGFP and SARS-CoV-2 HA-nsp3-4-V5 encoding plasmids. After 48 h cells were stained with HA-specific antibody and analyzed by confocal microscopy to visualize HA-nsp3 and AGPAT1-GFP. White square indicates the area shown as magnification on the right. **b** Schematics of the image-based approach used for the determination of SARS-CoV-2 replication and spread. In some experiments, infected cells were treated with drugs as indicated. **c** AGPATs are not involved in SARS-CoV-2 entry. Left panel: Huh7-Lunet control, AGPAT2 single (SKO) and AGPAT1/2 double (DKO) cells stably expressing human ACE2 were infected with VSVΔG-G and VSVΔG-S pseudoviruses encoding GFP. Twenty-four hours later, cells were fixed, nuclear DNA was stained with DAPI, and the percentage of GFP-positive cells was determined using CellProfiler. Normalized data from three biologically independent experiments are plotted. Right panel: Huh7-derived cells expressing human ACE2 were either pre- or post-incubated

with the AGPAT inhibitor CI976 (10 μM) for 4 h and infected with VSVΔG-G or VSVΔG-S pseudoviruses. Twenty-four hours later, cells were fixed, nuclear DNA was stained with DAPI, and the percentage of GFP-positive cells was determined using CellProfiler. Normalized data from three biologically independent experiments are plotted.

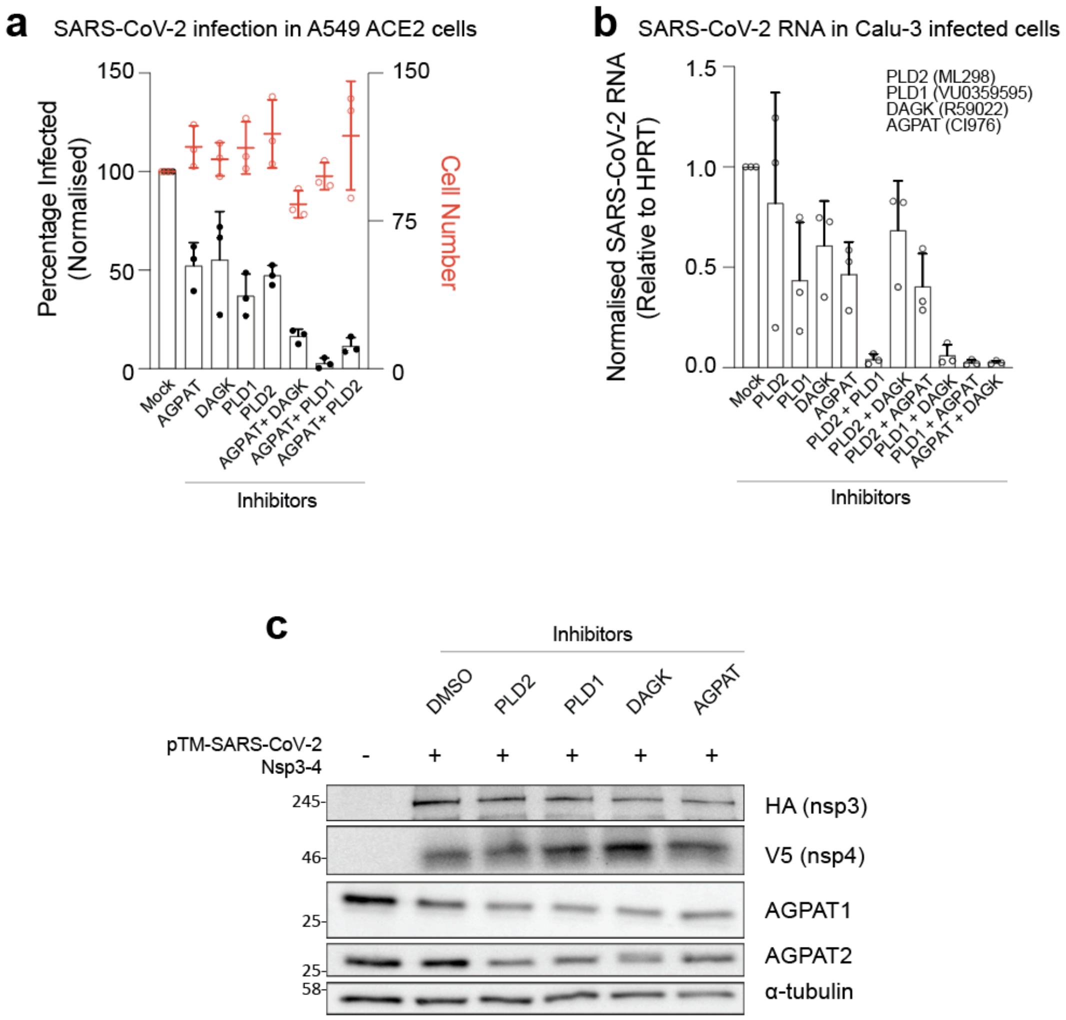

Supplementary Figure 10. Alternative PA production pathways contribute to SARS-CoV-2 replication. a A549 cells stably expressing ACE2 were infected with SARS-CoV-2 (MOI=5) and incubated with AGPAT, PLD1/2, or DAGK inhibitors, or given combinations thereof, using concentrations corresponding to IC50 values. Cells were fixed 24 h after infection and stained with a nucleocapsid-specific antibody, followed by immunofluorescence microscopy and CellProfiler based analysis. Data is normalized to the Mock, which on average has ~35% infected cells. Data are represented as mean ± SD from 3 biologically independent experiments. **b** Calu-3 cells were infected with SARS-CoV-2 (MOI=12) for 2 h, followed by incubation with AGPAT, PLD1/2, or DAGK inhibitors using concentrations corresponding to IC50 values. Total RNA was harvested 6 h after infection and SARS-CoV-2 RNA was quantified relative to cellular HPRT mRNA using RT-qPCR. Higher MOI than in (a) was used since first, unbound virus was washed away after 2 h and second, viral replication was measured at 6 h rather than 24 h after infection. Data are represented as mean ± SD from 3 biologically independent experiments. **c** PA pathway inhibitors do not affect SARS-CoV-2 nsp3-4 expression or processing. Huh7-Lunet/T7 cells were transfected with plasmid pTM-nsp3-4-2A-mNG encoding SARS-CoV-2 HA-nsp3-4-V5 and fluorescent NeonGreen. After 4 h, inhibitors specified on the top were added. Twenty-four hours later, total cell lysates were collected and expression levels of indicated proteins were analyzed by western blotting. α-tubulin served as loading control. These data belong to the results shown in Fig. 8c. A representative result of three biologically independent experiments is shown.

Supplementary Tables

Supplementary Table 1. Plasmids used in this study

Plasmid Name	Backbone	Epitope tag/ reporter	Selection (E.coli/mammalian cell)	Reference
pFK-JcR2a-δg (JcR2a)	pFK, derived from pBR322	*Renilla* Luciferase	Amp	[6]
pFK-Jc1-δg (Jc1),	pFK, derived from pBR322		Amp	[7]
pFK_i389LucNS3-3′_JFH1_δg (genotype 2a)	pFK, derived from pBR322	*Firefly* Luciferase	Amp	[8]
pFK_i389Luc_NS3-3′JFH1δg (genotype 2a) (NS5A-mcherry)	pFK, derived from pBR322	NS5A-mCherry/*Firefly* Luciferase	Amp	[4]
pTM NS3-5B	pTM1-2		Amp	[9]
pTM NS3-5B (NS5A-mcherry)	pTM1-2	NS5A-mCherry	Amp	[4]
pWPI-EGFP-CT	pWPI	EGFP	Amp/Blasti	This study.
pWPI-EGFP-NT	pWPI	EGFP	Amp/Blasti	This study.
pWPI-mCherry-NT	pWPI	mCherry	Amp/Blasti	This study.
pWPI-AGPAT1-EGFP	pWPI-EGFP	AGPAT1-EGFP	Amp/Blasti	This study.
pWPI-AGPAT2-EGFP	pWPI-EGFP	AGPAT2-EGFP	Amp/Blasti	This study.
pWPI-EGFP-2xPABD	pWPI-EGFP	EGFP-2xPABD (Spo20p)	Amp/Blasti	This study.
pWPI-EGFP-2xPABD_4E	pWPI-EGFP	EGFP-2xPABD_4E (Spo20p)	Amp/Blasti	This study.
pGEX-PABD	pGEX-6P-1	GST-1xPABD (Spo20p)	Amp	This study.
pGEX-PABD_4E	pGEX-6P-1	GST-1xPABD_4E (Spo20p)	Amp	This study.
pWPI-mCherry-Parkin	pWPI-mCherry	mCherry-Parkin	Amp/Blasti	This study.
pWPI-T7-Zeo	pWPI		Amp/Zeo	[10]
pCMV-dR8.91	pCMV		Amp	kind gift from Dr. Didier Trono
pMD2.G			Amp	kind gift from Dr. Didier Trono
lentiCRISPR v2	lentiCRISPR v2		Amp/Puro	Addgene
lentiCRISPR_blasticidin	lentiCRISPR v2		Amp/Blasti	[11]
lentiCRISPR_AGPAT1 #7	lentiCRISPR v2		Amp/Puro	This study.

lentiCRISPR_AGPAT1 #8	lentiCRISPR v2		Amp/Puro	This study.
lentiCRISPR_AGPAT2 #1	lentiCRISPR_blasticidin		Amp/Blasti	This study.
lentiCRISPR_AGPAT2 #2	lentiCRISPR_blasticidin		Amp/Blasti	This study.
pWPI-AGPAT1_sgRNA-resistant_wild-type (WT)	pWPI		Amp/Blasti	This study.
pWPI-AGPAT1_sgRNA-resistant_H104A, D109N (M1)	pWPI		Amp/Blasti	This study.
pWPI-AGPAT1_sgRNA-resistant_E178Q, R181A (M2)	pWPI		Amp/Blasti	This study.
pWPI-AGPAT2_sgRNA-resistant_wild-type (WT)	pWPI		Amp/Puro	This study.
pWPI-AGPAT2_sgRNA-resistant_H98A, D103N (M1)	pWPI		Amp/Puro	This study.
pWPI-AGPAT2_sgRNA-resistant_E172Q, R175A (M2)	pWPI		Amp/Puro	This study.
pTM-EGFP-PABD-Raf1_WT	pTM1-2	EGFP-PABD_WT (Raf1)	Amp	This study
pTM-EGFP-PABD-Raf1_4E	pTM1-2	EGFP-PABD_4E (Raf1)	Amp	This study
pWPI-EGFP-PABD-Raf1_WT	pWPI	EGFP-PABD_WT (Raf1)	Amp/Blasti	This study
pWPI-EGFP-PABD-Raf1_4E	pWPI	EGFP-PABD_4E (Raf1)	Amp/Blasti	This study
pcDNA3.1-SARS-CoV-2_HA-Nsp3-4-V5	pcDNA3.1	HA-Nsp3 Nsp4-V5	Amp	This study
pTM SARS-CoV-2_HA-Nsp3-4-V5 (IRES-mNG)	pTM1-2	mNeonGreen HA-Nsp3 Nsp4-V5	Amp	This study
pCMV3-SARS-CoV-2-Spike-codon-optimized-dCTD3	pCMV3		Amp/Hygro	This study

Amp, ampicillin; Blasti, blasticidin; Zeo, zeocine; Puro, puromycin; Hygro, Hygromycin B.

Supplementary Table 2. Reagents and resources used in this study

Reagents or Resources	Source	Identifier
DAPI	MoBiTec	MFPCCFA-211
Lipidtox	Thermo Fisher Scientific	H34477
BODIPY493/503	Thermo Fisher Scientific	D3922
Oleic acid-BSA	Sigma Aldrich	O3008
Mifepristone	Sigma Aldrich	M8046-500MG
Fluoromount-G	Southern Biotech	0100-01
Phorbol 12-myristate 13-acetate (PMA)	Sigma Aldrich	P8139
Bafilomycin A1 (BafA1)	Sigma Aldrich	B1793
Valinomycin (Val)	Sigma Aldrich	V0627
TransIT-LT1 transfection reagent	Mirus Bio	MIR 2305
In-Fusion HD Cloning Plus	TAKARA Bio	638909
NEB Hi-Fi Assembly Kit	New England Biolabs Inc	E2621S
Platinum™ SuperFi™ PCR Master Mix	Thermo Fisher Scientific	12358010
IPTG	Thermo Fisher Scientific	15529019
Slide-A-Lyzer Dialysis Cassettes, 7K MWCO	Thermo Fisher Scientific	66370
streptolysin O (SLO)	Sigma Aldrich	SAE0089
Creatine Kinase (CK)	Sigma Aldrich	CK-RO, 10127566001
Creatine phosphate	Sigma Aldrich	CRPHO-RO, 10621714001
Guanosine 5′-triphosphate sodium salt hydrate	Sigma Aldrich	G8877
Pierce Anti-HA Magnetic Beads	Thermo Fisher Scientific	88836
Pierce GST Spin Purification Kit	Thermo Fisher Scientific	16107
CellTiter-Glo Luminescent Cell Viability Assay	Promega	G7570
ECL plus reagent	Perkin Elmer Inc.	NEL104001EA
DTT	Sigma Aldrich	D0632
Glycyl Glycin	Sigma Aldrich	G3915
ATP	Sigma Aldrich	A2383
D-Luciferin	PJK GmbH	102111
Coelenterazine	PJK GmbH	102161
Paraformaldehyde	Sigma Aldrich	158127
25% Glutaraldehyde	Electron Microscopy Science	16220
16% Paraformaldehyde Aqueous Solution	Electron Microscopy Science	15710
4% Osmium Tetroxide	Electron Microscopy Science	19150
ML298	Sigma Aldrich	SML1077
VU0359595	Sigma Aldrich	SML0566
FIPI Hydrochloride Hydrate	Sigma Aldrich	F5807

R59022	Sigma Aldrich	D5919

Supplementary Table 3. Antibodies used in this study

Primary antibody	Source	Identifier
rabbit anti-HCV NS4B polyclonal antibody	[12]	-
mouse anti-HCV NS5A monoclonal antibody	[12]	-
rabbit anti-AGPAT1 polyclonal antibody	Atlas Antibodies	HPA073355
rabbit anti-AGPAT2 monoclonal antibody	Cell Signaling	14937
mouse anti-alpha-tubulin monoclonal antibody	Sigma Aldrich	T5168
mouse anti-beta-actin monoclonal antibody	Sigma Aldrich	A5441
rabbit anti-LC3 polyclonal antibody	MBL	PM036
mouse anti-HA monoclonal antibody	Sigma Aldrich	H3663
rabbit anti-HA polyclonal antibody	Thermo Fisher Scientific	PA1-985
mouse anti-V5 monoclonal antibody	GeneTex	GTX628529
rabbit anti-V5 polyclonal antibody	GeneTex	GTX117997
mouse anti-GST monoclonal antibody	Santa Cruz	sc-138
**mouse anti-Nucleocapsid antibody**	Sino Biological	40143-V08B
mouse anti-GFP antibody	Clontech	632381
mouse anti-Flag antibody	Sigma Aldrich	F1804
mouse anti-VSV-G antibody	Produced in I1-Hybridoma cells	-

Secondary antibody	Source	Identifier
Goat anti–rabbit IgG-HRP	Sigma Aldrich	A6154
Goat anti–mouse IgG-HRP	Sigma Aldrich	A4416
Alexa Fluor 488 donkey anti-rabbit IgG	Thermofisher	A-21206
Alexa Fluor 488 donkey anti-mouse IgG	Thermofisher	A-21202
Alexa Fluor 488 donkey anti-mouse IgG2a	Thermofisher	A-21131
Alexa Fluor 568 donkey anti-rabbit IgG	Thermofisher	A-10042
Alexa Fluor 568 donkey anti-mouse IgG	Thermofisher	A-10037
Alexa Fluor 568 donkey anti-mouse IgG1	Thermofisher	A-21124

Supplementary Table 4. Cell lines used in this study

Descriptor	Parental cell line	resistance	reference
HEK-293T	HEK-293T		[13]
Huh7.5	Huh7.5		[14]
Huh7-Lunet	Huh7-Lunet		[15]
Huh7.5/Fluc	Huh7.5	Geneticin	[6]
Huh7-Lunet/T7	Huh7-Lunet	Zeocin	[9]
Huh7-Lunet/CD81H	Huh7-Lunet	Geneticin	[16]
Huh7-Lunet/subgenomic replicon [HCV wt]	Huh7-Lunet	Geneticin	[12]
Huh7-Lunet/subgenomic replicon (sg4B HA31R) [HCV 4BHA]	Huh7-Lunet	Geneticin	[12]
Huh7-Lunet/calnexin^{HA} [CNX^{HA}]	Huh7-Lunet	Geneticin	[12]
Huh7-Lunet/AGPAT1-EGFP	Huh7-Lunet	Blasticidin	This study.
Huh7-Lunet/AGPAT2-EGFP	Huh7-Lunet	Blasticidin	This study.
Huh7.5/AGPAT1_sgRNA-resistant_wild-type (WT)	Huh7.5	Blasticidin	This study.
Huh7.5/AGPAT1_sgRNA-resistant_H104A, D109N (M1)	Huh7.5	Blasticidin	This study.
Huh7.5/AGPAT1_sgRNA-resistant_E178Q, R181A (M2)	Huh7.5	Blasticidin	This study.
Huh7.5/AGPAT2_sgRNA-resistant_wild-type (WT)	Huh7.5	Puromycin	This study.
Huh7.5/AGPAT2_sgRNA-resistant_H98A, D103N (M1)	Huh7.5	Puromycin	This study.
Huh7.5/AGPAT2_sgRNA-resistant_E172Q, R175A (M2)	Huh7.5	Puromycin	This study.
Huh7-Lunet/T7/mCherry-Parkin	Huh7-Lunet	Zeocin, Blasticidin	This study.
Huh7-Lunet/CD81H/mCherry-Parkin	Huh7-Lunet	Geneticin	This study.
Huh7-Lunet/T7/Control KO	Huh7-Lunet	Zeocin, Puromycin	This study.
Huh7-Lunet/T7/AGPAT DKO	Huh7-Lunet	Zeocin, Blasticidin, Puromycin	This study.
Huh7-Lunet/T7/ACE2	Huh7-Lunet	Zeocin, Blasticidin, Puromycin Neomycin	This study.
A549-ACE2	A549	Neomycin	[5]
Calu-3	Calu-3		[5]

BHK-G43	BHK-21	Hygromycin B Zeocin	[17]
I1-Hybridoma	Primary murine spleen cells and Sp2/0-Ag14 mouse myeloma cells		ATCC: CRL-2700™

Supplementary Table 5. Vectors and guide RNAs used to generate AGPAT KO cells

Expression plamid	Target gene	Guide RNA
lentiCRISPR_AGPAT1 #7	AGPAT1	5-GGGGCTGCAGAACCACAGGG-3
lentiCRISPR_AGPAT1 #8	AGPAT1	5'-GAGGGAACGAGAAACCACAA-3'
lentiCRISPR_AGPAT2 #1	AGPAT2	5'-CGCGGCCGAGTTCTACGCCA-3'
lentiCRISPR_AGPAT2 #2	AGPAT2	5'-CTTTTACGGGCTCCGCTTCG-3'

References

1. Ritchie ME*, et al.* limma powers differential expression analyses for RNA-sequencing and microarray studies. *Nucleic Acids Res* **43**, e47 (2015).

2. Shannon P*, et al.* Cytoscape: a software environment for integrated models of biomolecular interaction networks. *Genome Res* **13**, 2498-2504 (2003).

3. Bindea G*, et al.* ClueGO: a Cytoscape plug-in to decipher functionally grouped gene ontology and pathway annotation networks. *Bioinformatics* **25**, 1091-1093 (2009).

4. Lee JY*, et al.* Spatiotemporal Coupling of the Hepatitis C Virus Replication Cycle by Creating a Lipid Droplet- Proximal Membranous Replication Compartment. *Cell Rep* **27**, 3602-3617 e3605 (2019).

5. Cortese M*, et al.* Integrative Imaging Reveals SARS-CoV-2-Induced Reshaping of Subcellular Morphologies. *Cell Host Microbe* **28**, 853-866 e855 (2020).

6. Reiss S*, et al.* Recruitment and activation of a lipid kinase by hepatitis C virus NS5A is essential for integrity of the membranous replication compartment. *Cell Host Microbe* **9**, 32-45 (2011).

7. Pietschmann T*, et al.* Construction and characterization of infectious intragenotypic and intergenotypic hepatitis C virus chimeras. *Proc Natl Acad Sci U S A* **103**, 7408-7413 (2006).

8. Lohmann V, Korner F, Koch J, Herian U, Theilmann L, Bartenschlager R. Replication of subgenomic hepatitis C virus RNAs in a hepatoma cell line. *Science* **285**, 110-113 (1999).

9. Backes P*, et al.* Role of annexin A2 in the production of infectious hepatitis C virus particles. *J Virol* **84**, 5775-5789 (2010).

10. Fuerst TR, Niles EG, Studier FW, Moss B. Eukaryotic transient-expression system based on recombinant vaccinia virus that synthesizes bacteriophage T7 RNA polymerase. *Proc Natl Acad Sci U S A* **83**, 8122-8126 (1986).

11. Neufeldt CJ*, et al.* ER-shaping atlastin proteins act as central hubs to promote flavivirus replication and virion assembly. *Nat Microbiol*, (2019).

12. Paul D, Hoppe S, Saher G, Krijnse-Locker J, Bartenschlager R. Morphological and biochemical characterization of the membranous hepatitis C virus replication compartment. *J Virol* **87**, 10612-10627 (2013).

13. Graham FL, Smiley J, Russell WC, Nairn R. Characteristics of a human cell line transformed by DNA from human adenovirus type 5. *J Gen Virol* **36**, 59-74 (1977).

14. Blight KJ, McKeating JA, Rice CM. Highly permissive cell lines for subgenomic and genomic hepatitis C virus RNA replication. *J Virol* **76**, 13001-13014 (2002).

15. Friebe P, Boudet J, Simorre JP, Bartenschlager R. Kissing-loop interaction in the 3' end of the hepatitis C virus genome essential for RNA replication. *J Virol* **79**, 380-392 (2005).

16. Koutsoudakis G*, et al.* Characterization of the early steps of hepatitis C virus infection by using luciferase reporter viruses. *J Virol* **80**, 5308-5320 (2006).

17. Berger Rentsch M, Zimmer G. A vesicular stomatitis virus replicon-based bioassay for the rapid and sensitive determination of multi-species type I interferon. *PLoS One* **6**, e25858 (2011).

Source Blots

Fig. 1d

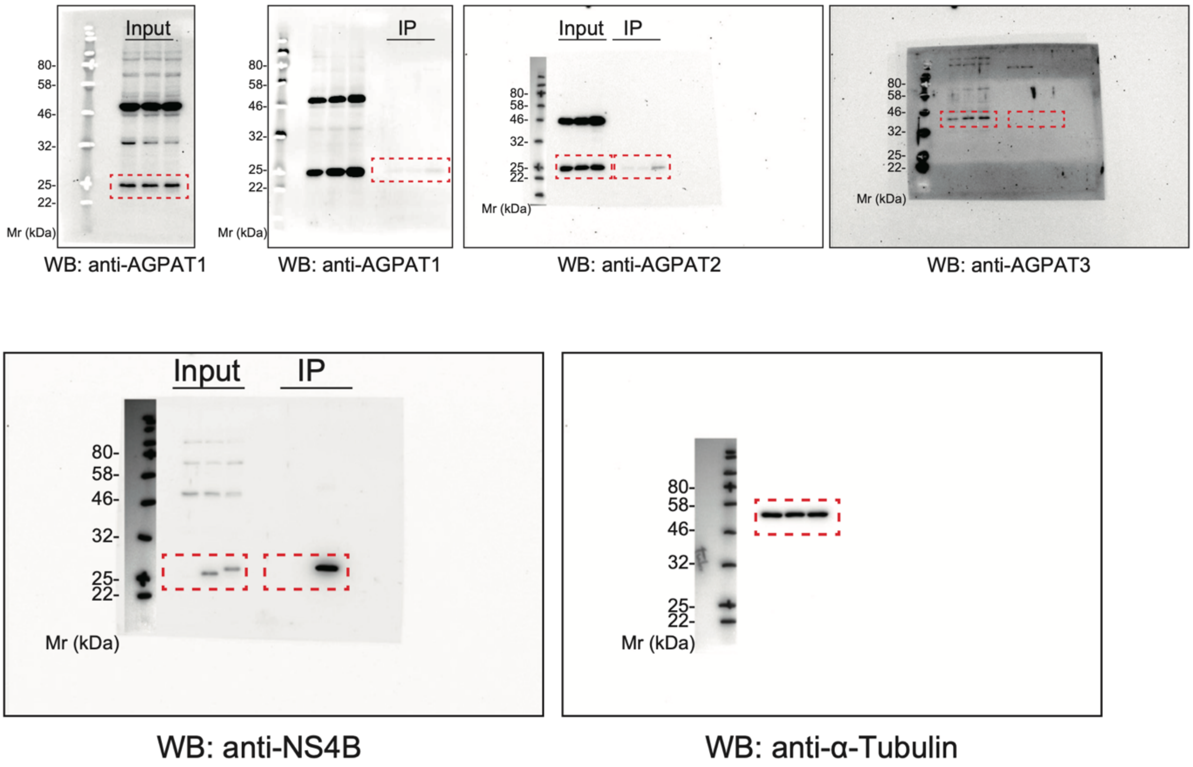

Fig. 1f

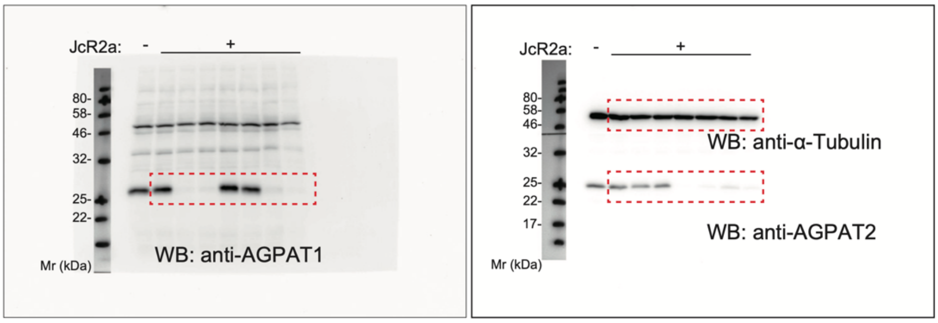

Fig. 1g

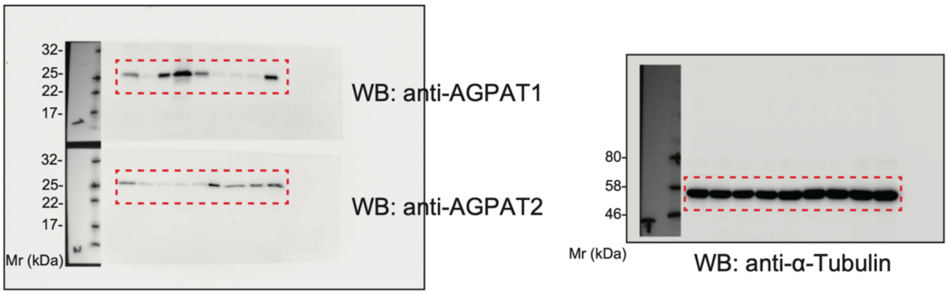

Fig. 2c

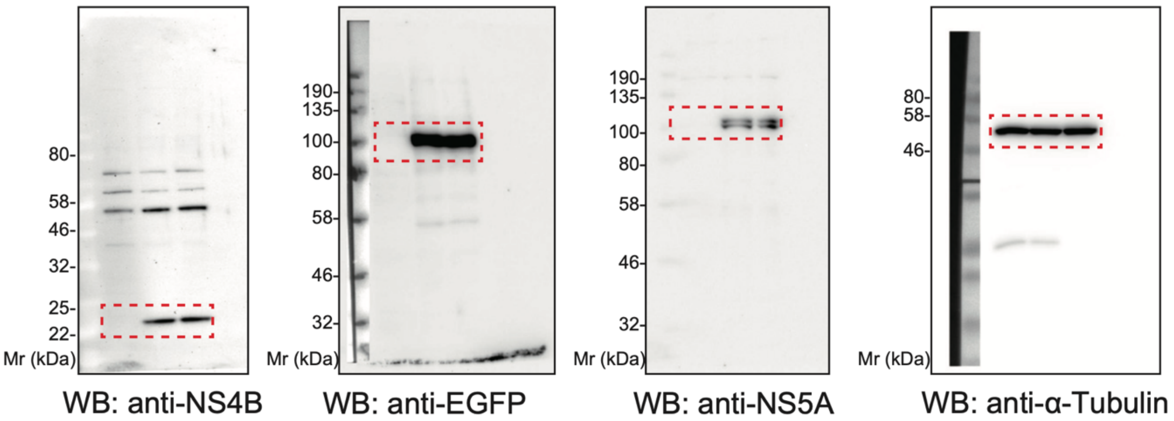

Fig. S1a

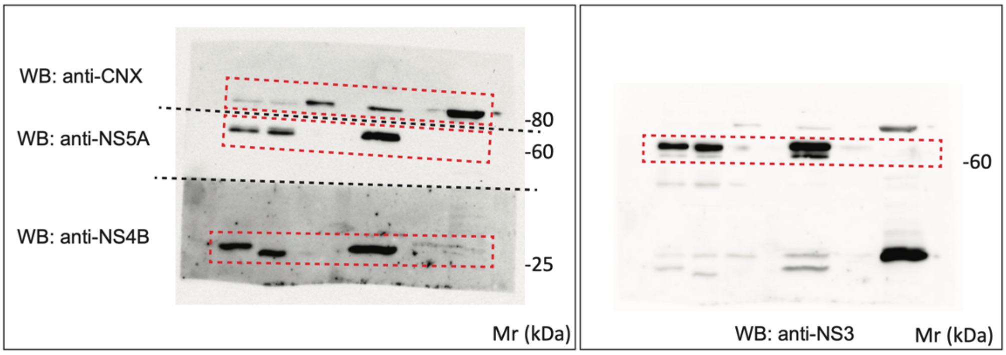

Fig. S3a

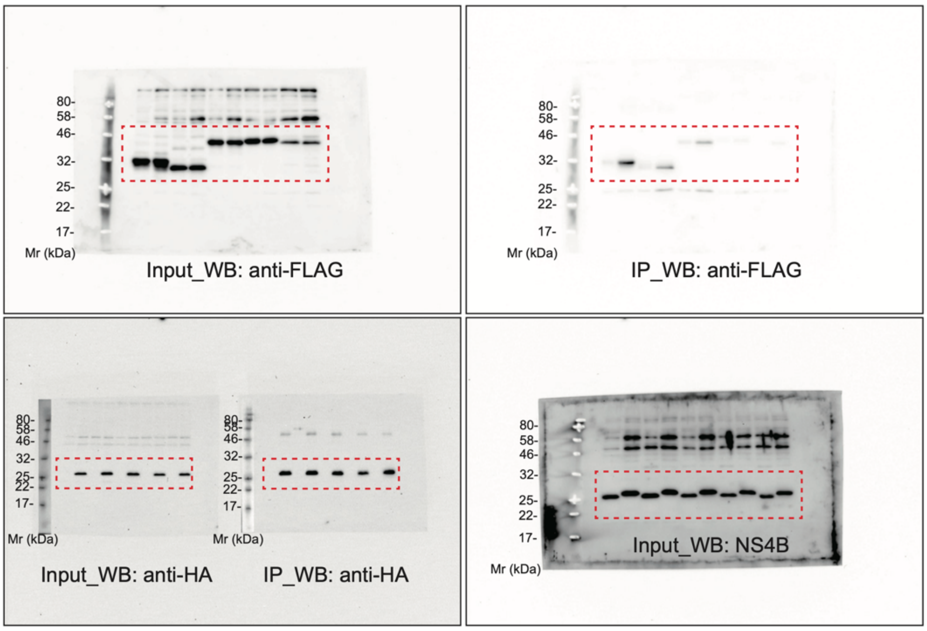
80-
58-
46-
32-
25-
22-
17-
Mr (kDa)
Input_WB: anti-FLAG
IP_WB: anti-FLAG
Input_WB: anti-HA
IP_WB: anti-HA
Input_WB: NS4B

Fig. S8c

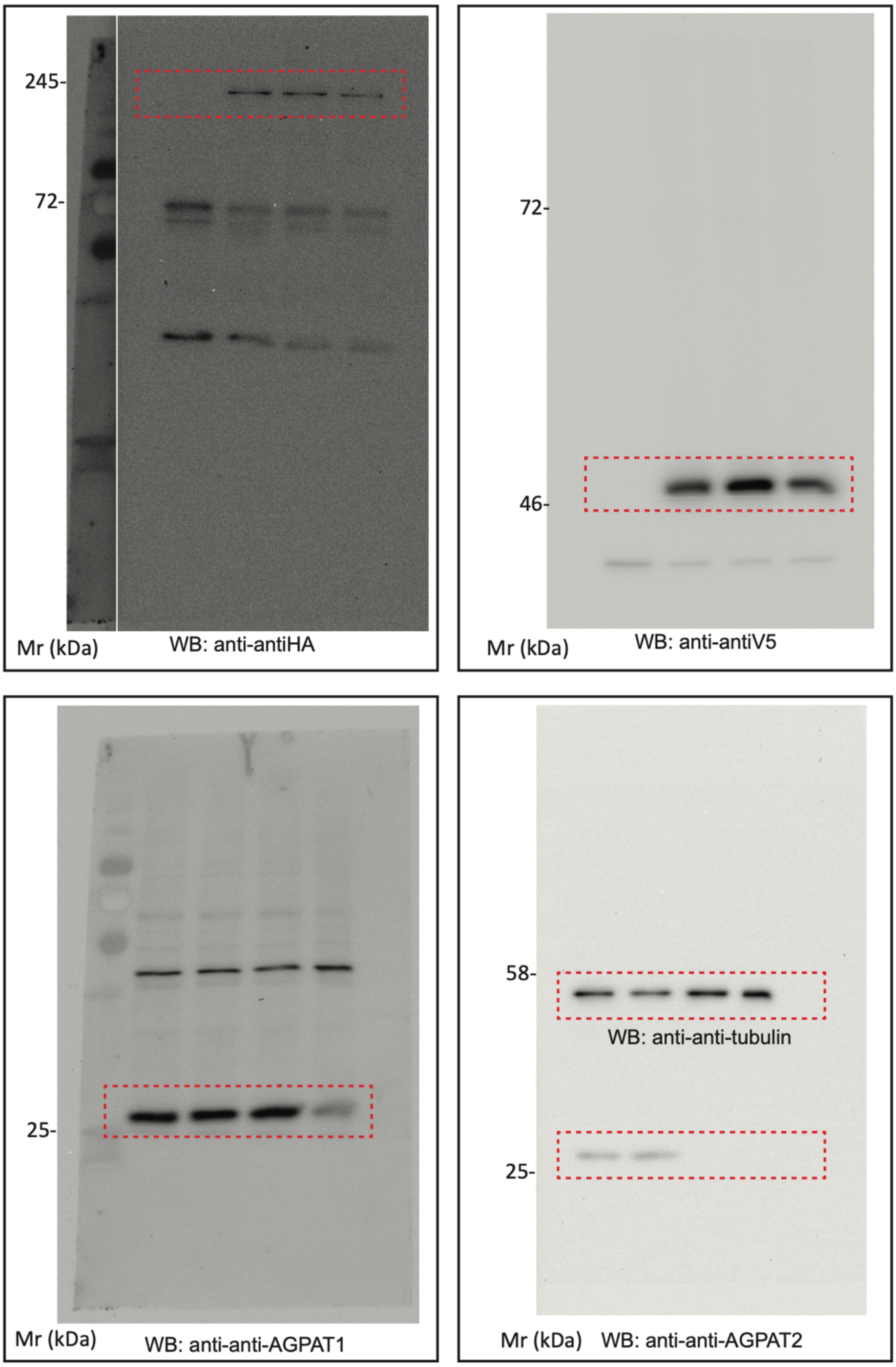
245-
72-
Mr (kDa)
WB: anti-antiHA
72-
46-
Mr (kDa)
WB: anti-antiV5
25-
Mr (kDa)
WB: anti-anti-AGPAT1
58-
WB: anti-anti-tubulin
25-
Mr (kDa)
WB: anti-anti-AGPAT2

Fig. S10c

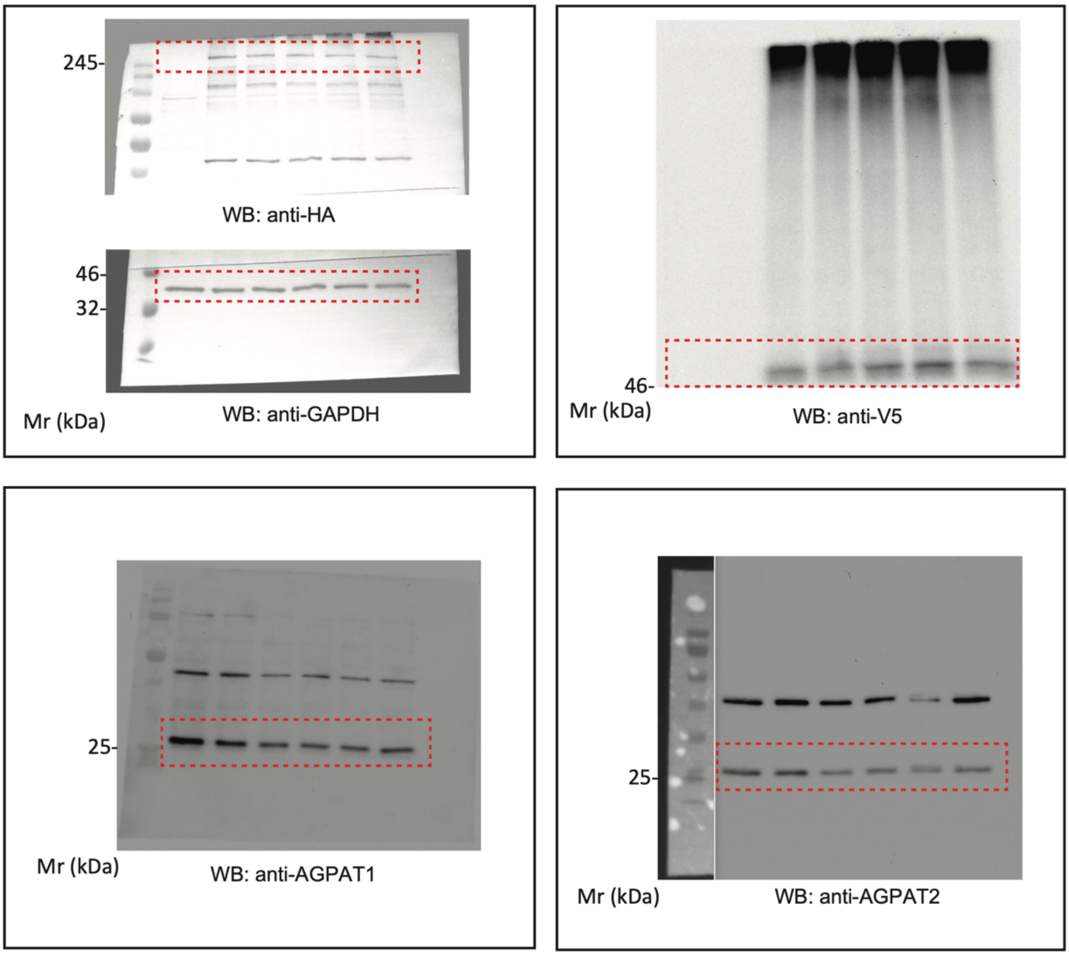

245-
WB: anti-HA
46-
32-
Mr (kDa)
WB: anti-GAPDH
46-
Mr (kDa)
WB: anti-V5
25-
Mr (kDa)
WB: anti-AGPAT1
25-
Mr (kDa)
WB: anti-AGPAT2
