# Supplementary material for: Convergent use of phosphatidic acid for hepatitis C virus and SARS-CoV-2 replication organelle formation
Source: Nat Commun. 2021 Dec 14;12:7276. doi: 10.1038/s41467-021-27511-1 (PMC8671429; doi:10.1038/s41467-021-27511-1)
Supplement: Supplementary file 3 — Description of Additional Supplementary Information [file 41467_2021_27511_MOESM3_ESM.pdf]

Description of Additional Supplementary Files

File Name: Supplementary Data 1

Description: Proteome analysis. LC-MS/MS RAW files obtained for untagged WT-, NS4BA- and CNXassociated membrane samples were analyzed with Proteome Discoverer 2.2 (PD2.2) using 1% false discoverer rate (FDR) on peptide-spectrum-match (PSM) and protein level. Label-free quantitation with 'match between runs' function was used for calculating abundance values (see PD2.2 MS data analysis). Results were used for 'Significance Analysis of INTeractome' (SAINT) using untagged NS4B as technical negative control (see NS4B_saint_result and CNX_saint_result). All proteins considered as interactors by SAINT (AvgP>0.95) of at least one of the baits (NS4B or CNX) were normalized to equal total abundance in each sample and analyzed for differential abundance between the NS4B and CNX pulldowns using the limma R software package (see limma_output). NS4B significantly enriched proteins (see limma_NS4BA_enriched) were used to generate functional networks utilizing the ClueGO v2.5.5 app embedded in Cytoscape 3.7.2 (see ClueGO-NS4BA).

File Name: Supplementary Data 2

Description: SiRNA screening. The following information is listed in different excel sheets: the "control siRNAs" sheet provides siRNA sequences and product numbers of siRNAs that were used in the screen; the "siRNA list_Silencer select" sheet provides all target genes and siRNAs used in the screen; the "Plate Layout" sheet shows the layout design for each plate that was used in the screen (three different siRNAs per target gene; control siRNAs were randomly placed in different plates to avoid position effects); the "A) Result_raw counts" sheet lists the luciferase counts after subtraction of the background values; in the "B) Result_sorted by z-score" sheet data are sorted by z-score; the "C) Result_gene_z-score" sheet lists the genes according to z-score after statistical analysis using data from three different siRNAs and 5 repetitions.

File Name: Supplementary Data 3

Description: Lipidome analysis. Raw data set of lipidome analysis of HCV-induced DMVs. Quantified lipids are listed including concentrations (in µM) of all individual lipid species.
