# Supplementary material for: Convergent use of phosphatidic acid for hepatitis C virus and SARS-CoV-2 replication organelle formation
Source: Nat Commun. 2021 Dec 14;12:7276. doi: 10.1038/s41467-021-27511-1 (PMC8671429; doi:10.1038/s41467-021-27511-1)
Supplement: Supplementary file 7 — Reporting Summary [file 41467_2021_27511_MOESM7_ESM.pdf]

nature research

Corresponding author(s): Ralf Bartenschlager

Last updated by author(s): Nov 1, 2021

Reporting Summary

Nature Research wishes to improve the reproducibility of the work that we publish. This form provides structure for consistency and transparency in reporting. For further information on Nature Research policies, see our Editorial Policies and the Editorial Policy Checklist.

Statistics

For all statistical analyses, confirm that the following items are present in the figure legend, table legend, main text, or Methods section.

n/a	Confirmed	
☐	☒	The exact sample size (n) for each experimental group/condition, given as a discrete number and unit of measurement
☐	☒	A statement on whether measurements were taken from distinct samples or whether the same sample was measured repeatedly
☐	☒	The statistical test(s) used AND whether they are one- or two-sided<br>*Only common tests should be described solely by name; describe more complex techniques in the Methods section.*
☒	☐	A description of all covariates tested
☒	☐	A description of any assumptions or corrections, such as tests of normality and adjustment for multiple comparisons
☐	☒	A full description of the statistical parameters including central tendency (e.g. means) or other basic estimates (e.g. regression coefficient) AND variation (e.g. standard deviation) or associated estimates of uncertainty (e.g. confidence intervals)
☐	☒	For null hypothesis testing, the test statistic (e.g. F, t, r) with confidence intervals, effect sizes, degrees of freedom and P value noted<br>*Give P values as exact values whenever suitable.*
☒	☐	For Bayesian analysis, information on the choice of priors and Markov chain Monte Carlo settings
☒	☐	For hierarchical and complex designs, identification of the appropriate level for tests and full reporting of outcomes
☒	☐	Estimates of effect sizes (e.g. Cohen's d, Pearson's r), indicating how they were calculated

Our web collection on statistics for biologists contains articles on many of the points above.

Software and code

Policy information about availability of computer code

Data collection	- High-thoughput image data collection was performed using Zeiss Cell Discoverer 7 and Nikon Andor Spinning disk high-throughput confocal microscopes.<br>- Confocal images were collected with Leica SP8 microscope.
Data analysis	- All image based segmentation was performed in CellProfiler software, except Supplementary Fig 3(D and E), which were analysed with ImageJ.<br>- GraphPad Prism software was used for plotting of scatter plot and statistical tests.<br>- CellProfiler Analyst was used to classify cells into groups of infected or non-infected cells, or other classes defined in the figures or legends.

For manuscripts utilizing custom algorithms or software that are central to the research but not yet described in published literature, software must be made available to editors and reviewers. We strongly encourage code deposition in a community repository (e.g. GitHub). See the Nature Research guidelines for submitting code & software for further information.

Data

Policy information about availability of data

All manuscripts must include a data availability statement. This statement should provide the following information, where applicable:

- Accession codes, unique identifiers, or web links for publicly available datasets
- A list of figures that have associated raw data
- A description of any restrictions on data availability

- The scripts, training set and sample images to train the classifier for Figs 5b, 7b, and 8b are deposited at Mendeley Data (https://doi.org/10.17632/b6hdc96ks5.1).

Field-specific reporting

Please select the one below that is the best fit for your research. If you are not sure, read the appropriate sections before making your selection.

- [x] Life sciences
- [ ] Behavioural & social sciences
- [ ] Ecological, evolutionary & environmental sciences

For a reference copy of the document with all sections, see nature.com/documents/nr-reporting-summary-flat.pdf

Life sciences study design

All studies must disclose on these points even when the disclosure is negative.

Sample size	- Sample size was not pre-determined.<br>- No statistical methods were used to predetermine sample sizes.<br>- Sample sizes are stated in the figure legends and methods section when applicable.
Data exclusions	No data was excluded in any of the experiments.
Replication	Experiments were biologically and experimentally reproduced on separate days, separate batches of drugs and different passages of the cell lines to confirm replication of the effect. The exact number of biological repetitions are stated in the figure legends for each figure sub-section.
Randomization	Covariates are not relevant to our study. For Supplementary Fig. 7c and 7d, random sampling of the data points were done to keep the sample size constant between non-treated and drug treated samples. Additional analysis were done to confirm that the trends and results were the same using all cells.
Blinding	Blinding was not done during data acquisition or analysis since in most cases data is high-throughput in nature and analysis were done in an automated manner. Analysis using softwares minimized the occurrence of any bias since most of the analysis were done in an automated manner.

Reporting for specific materials, systems and methods

We require information from authors about some types of materials, experimental systems and methods used in many studies. Here, indicate whether each material, system or method listed is relevant to your study. If you are not sure if a list item applies to your research, read the appropriate section before selecting a response.

Materials & experimental systems

n/a	Involved in the study	
[ ]	[x]	Antibodies
[ ]	[x]	Eukaryotic cell lines
[x]	[ ]	Palaeontology and archaeology
[x]	[ ]	Animals and other organisms
[x]	[ ]	Human research participants
[x]	[ ]	Clinical data
[x]	[ ]	Dual use research of concern

Methods

n/a	Involved in the study	
[x]	[ ]	ChIP-seq
[x]	[ ]	Flow cytometry
[x]	[ ]	MRI-based neuroimaging

Antibodies

Antibodies used	Primary antibodies for immunofluorescence:<br>- NS4B - (rabbit, source: doi:10.1128/JVI.01370-13, 2013).<br>- NS5A - (rabbit, source: doi:10.1128/JVI.01370-13, 2013).<br>- GFP (mouse, Clontech #632381).<br>- HA - (mouse, Sigma Aldrich H3663), (rabbit, Thermo Scientific, PA1-985).<br>- V5 - (mouse, GeneTex #GTX628529), (rabbit, GeneTex #GTX117997).<br>- AGPAT1 - (rabbit, Atlas Antibodies #HPA073355).<br>- AGPAT2 - (rabbit, Cell Signaling #14937).<br>- GAPDH - (Santa Cruz Biotech #365062).<br>- alpha-tubulin - (mouse, Sigma Aldrich #T5168)<br>- beta-actin - (mouse, Sigma Aldrich #A5441)<br>- LC3 antibody - (rabbit, MBL #M162-3)<br>- Flag - (mouse, Sigma Aldrich #F1804)<br>- GST - (mouse, Santa Cruz Biotech #138)<br>- Nucleocapsid (mouse, Sino Biological, #40143-V08B)

Detailed doc and references are provided in the Antibodies section of methods in tabular form.

Validation

- NS4B - (rabbit, source: doi:10.1128/JVI.01370-13, 2013). antibody was validated by detection of expected molecular weight NS4B immunoblot band in only HCV infected cells. Further, NS4B expression constructs showed the same bands at expected sizes.
- NS5A - (rabbit, source: doi:10.1128/JVI.01370-13, 2013). antibody was validated by detection of expected molecular weight NS5A immunoblot band in only HCV infected cells. Further, NS5A expression constructs showed the same bands at expected sizes.
- GFP antisera was validated by detection of the immoblot band only after transfection of GFP-fusion construct and at expected molecular weight. Further validation was done by titration of the expression of GFP-fused PA-sensor.
- HA antisera was validated by detection of the immunoflourescence signal at expected cellular compartment only after transfection of HA-fusion construct. Cross-validation by detection of the immoblot band only after transfection of HA-fusion construct and at expected molecular weight was done.
- V5 - V5 antisera was validated by detection of the immunoflourescence signal at expected cellular compartment only after transfection of V5-fusion construct.
- AGPAT1 was validated by the loss of immunoblot band consistent with molecular weight of AGPAT1 in transient and stable AGPAT1-KO Lunet cells, when compared with Wild-type cells.
- AGPAT2 was validated by the loss of immunoblot band consistent with molecular weight of AGPAT2 in stable AGPAT2-KO Lunet cells, when compared with Wild-type cells.
- LC3 antibody was validated by observing an increase in LC3 specific puncta in Lunet cells under starvation conditions.
- GST - (mouse, Santa Cruz Biotech #138) antibody was validated by detection of immunolot band consistent with the expected band size of the purified GFP-fused PA-sensor.
- Nucleocapsid antibody was validated by detecting the N-specific signal in SARS-CoV-2 infected cells by immunofluorescece, and by oberving the immunoblot band consistent with the molecular weight of viral nucleocapsid protein.
- Alpha-tubulin was validated by detection of expected molecular weight immunoblot band in total cell lysates resolved on polyacrylamide gel.
- Beta-actin was validated by detection of expected molecular weight immunoblot band in total cell lysates resolved on polyacrylamide gel

Eukaryotic cell lines

Policy information about cell lines

Cell line source(s)

- HEK-293T, source: doi:10.1099/0022-1317-36-1-59
- Huh7.5, source: doi:10.1128/jvi.76.24.13001-13014.2002
- Huh7-Lunet, source: doi:10.1128/JVI.79.1.380-392.2005
- Huh7.5/Fluc, source: doi:10.1016/j.chom.2010.12.002
- Huh7-Lunet/T7, source: doi:10.1128/JVI.02343-09
- Huh7-Lunet/CD81H, source: doi:10.1128/JVI.02460-05
- Huh7-Lunet/subgenomic replicon [HCV wt], source: doi:10.1128/JVI.01370-13
- Huh7-Lunet/subgenomic replicon (sg4B HA31R) [HCV 4BHA], source: doi:10.1128/JVI.01370-13
- Huh7-Lunet/calnexinHA [CNX-HA], source: doi:10.1128/JVI.01370-13
- Huh7-Lunet/AGPAT1-EGFP, source: This study
- Huh7-Lunet/AGPAT2-EGFP, source: This study
- Huh7.5/AGPAT1_sgRNA-resistant_wild-type (WT), source: This study
- Huh7.5/AGPAT1_sgRNA-resistant_H104A, D109N (M1), source: This study
- Huh7.5/AGPAT1_sgRNA-resistant_E178Q, R181A (M2), source: This study
- Huh7.5/AGPAT2_sgRNA-resistant_wild-type (WT), source: This study
- Huh7.5/AGPAT2_sgRNA-resistant_H98A, D103N (M1), source: This study
- Huh7.5/AGPAT2_sgRNA-resistant_E172Q, R175A (M2), source: This study
- Huh7-Lunet/T7/mCherry-Parkin, source: This study
- Huh7-Lunet/CD81H/mCherry-Parkin, source: This study
- Huh7-Lunet/T7/Control KO, source: This study
- Huh7-Lunet/T7/AGPAT DKO, source: This study
- Huh7-Lunet/T7/ACE2, source: This study
- A549-ACE2, source: doi:10.1016/j.chom.2020.11.003 (2020)
- Calu-3, source: doi:10.1016/j.chom.2020.11.003 (2020)

Detailed informations and references are provided in the Cell Lines section of methods in Table S4.

Authentication

The cell lines used were not authenticated.

Mycoplasma contamination

All the cell lines have been checked and tested negative for mycoplasma contamination

Commonly misidentified lines
(See ICLAC register)

No cell lines from the ICLAC register were used.
